# End-Stage Tibiotalar Osteoarthritis and Chronic Strontium Toxicity

**DOI:** 10.7759/cureus.16866

**Published:** 2021-08-04

**Authors:** Gianni Ricci, Andrew S Bae, Benjamin Catoe, Benjamin C Watson

**Affiliations:** 1 Orthopaedic Surgery Residency, Jack Hughston Memorial Hospital, Phenix City, USA; 2 Orthopaedic Surgery, The Hughston Foundation, Columbus, USA; 3 Orthopaedic Surgery, Hughston Clinic, Columbus, USA

**Keywords:** osteoarthritis, metallosis, strontium, tibiotalar, osteoporosis, metal toxicity, bilateral ankle pain, ankle deformity, tibiotalocalcaneal fusion

## Abstract

Metal toxicity due to environmental sources or orthopedic implants has been a primary focus in recent literature. Specifically, in orthopedics, total joint arthroplasty regarding metal-on-metal articulation with cobalt-chromium articulation has adverse local and systemic effects. In particular, strontium toxicity is less known metal toxicity that can cause many systemic effects such as severe osteoporosis. This is the first reported case of strontium toxicity and end-stage tibiotalar osteoarthritis.

We present a case of a 68-year-old female with bilateral ankle pain and deformity that were refractory to conservative measures, including physical therapy and anti-inflammatory medications. She was diagnosed with bilateral osteoarthritis and osteoporosis secondary to strontium toxicity by exclusion after extensive workup with a multi-disciplinary approach. The patient pursued conservative measures with ankle-foot orthosis, physical therapy, and anti-inflammatory medications. After the failure of conservative measures with over two years of follow-up, we recommended operative intervention to improve function and pain with staged bilateral tibiotalocalcaneal fusions utilizing an intramedullary device. Since she is moving out of state, she chose to pursue operative intervention at a different institution in order to establish long-term follow-up. The patient was placed on teriparatide for her osteoporosis secondary to strontium toxicity. Clinicians should be aware of strontium toxicity and its systemic effects and take a multi-disciplinary approach to treatment for optimal management.

## Introduction

Metal toxicity has been the primary focus of numerous research studies and case reports. The source of toxicity has been traced from environmental exposures to orthopedic implants, and while some cases demonstrate a definitive source for metal toxicity, some are purely speculative in nature. Cobalt and chromium toxicity has been the primary focus of research related to orthopedic implants leading to metal toxicity. However, other metals, which may be contained within implants, surrounding bone cement, and even medications can also lead to metal toxicity [1]. More specifically, the metal ion strontium can be absorbed and preferentially stored in the bones and teeth.

Strontium is chemically and physically similar to calcium and it was first introduced therapeutically for bone pain in the setting of metastatic prostate cancer in 1940 [2]. Low doses of strontium appear to be beneficial in bone health through the binding of hydroxyapatite crystals in bone. It has been shown to reduce adverse events such as fractures in osteoporotic patients as high as 31% by means of improved structural and mechanical properties of bone [3]. However, higher concentrations of strontium appear to be detrimental to the bone by preferentially binding to calcium and reducing the percentage of calcium deposited in bone [4]. Several studies demonstrated that chronically toxic levels of strontium can cause decreased bone mineral density (BMD) leading to significant osteoporosis. We report a rare case of bilateral end-stage tibiotalar osteoarthritis with significant varus deformity in a 68-year-old female with chronic strontium toxicity. We reviewed the literature using search terms of strontium toxicity, osteoporosis, and metallosis. After a review of the literature, this appears to be the first described case of bilateral end-stage tibiotalar osteoarthritis in a patient secondary to strontium toxicity.

## Case presentation

The patient is a 68-year-old Caucasian female who presented to our foot and ankle clinic for evaluation of bilateral ankle pain and deformity that continued to worsen over the course of five years. She noted the development of a significant limp leading to balance and coordination issues requiring the use of a cane for ambulatory assistance. She completed several years of physical therapy without significant improvement in function, deformity, or reduction in pain. She had been prescribed an ankle-foot orthosis (AFO), which provided minimal improvement and was subsequently discontinued. Her past medical history consisted of systemic metallosis with persistently elevated strontium levels (1,280 μg/L) beginning in 2011, roughly two years after undergoing a metal on metal right total hip arthroplasty. The components implanted were the Depuy Pinnacle Hip System comprised of cobalt-chromium alloy. Subsequently, she underwent a successful right total hip arthroplasty revision in 2017. However, her strontium levels remained elevated following the revision surgery. During this time, she had developed hypothyroidism, poor dentition with maxillary osteolysis, expressive aphasia, urinary frequency, vision difficulties, severe osteoporosis, macrocytic anemia secondary to vitamin B12, and iron deficiencies. She was receiving care with adequate follow-up from endocrinology, hematology, cardiology, and primary care for the management of her chronic metal toxicity and related complications. After evaluation and extensive workup, endocrinology attributed her systemic symptoms and complications to chronic metal toxicity. They also attributed her severe osteoporosis to her chronic strontium toxicity.

Physical examination of the patient revealed bilateral tibiotalar edema with a flexible hindfoot varus deformity. She was tender to palpation along the medial and lateral aspects of her ankles. Her active range of motion (AROM) of the left ankle was restricted to 5° of dorsiflexion and 0° of inversion/eversion with normal plantarflexion, and her right ankle was restricted to 10° of dorsiflexion and 5° of inversion/eversion with normal plantarflexion. Ligamentous laxity was appreciated with talar tilt and anterior drawer testing with the ankle at neutral and 20° of plantarflexion bilaterally. She was otherwise motor and sensory intact with a well-perfused limb with no overlying skin changes.

**Figure 1:**
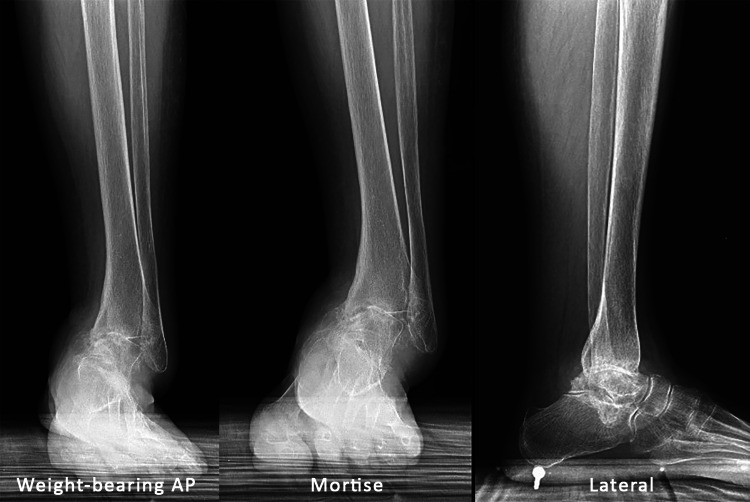
Left ankle: AP, mortise, and lateral radiographs Weight-bearing anteroposterior (AP), mortise, and lateral of the left ankle demonstrating significant tibiotalar and subtalar osteoarthritis with varus hindfoot and talar tilt.

Anteroposterior (AP), lateral, and mortise weight-bearing radiographs of the left ankle demonstrated significant tibiotalar and subtalar joint osteoarthritis with varus hindfoot deformity and talar tilt (Figure 1).

**Figure 2:**
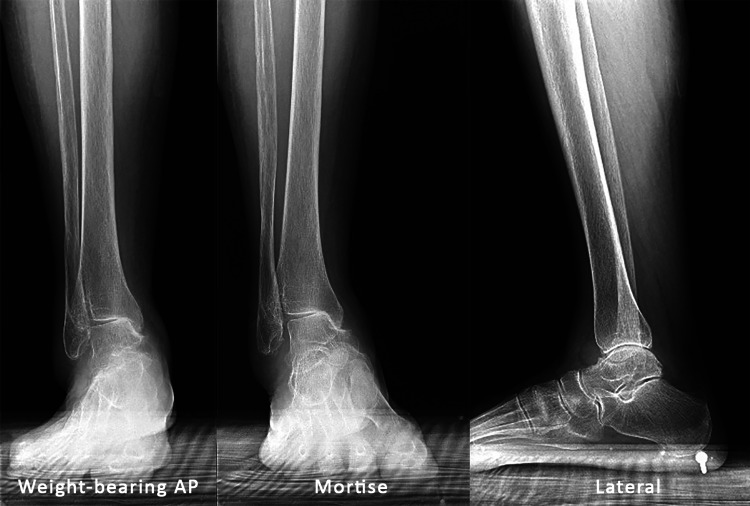
Right ankle: AP, mortise, and lateral radiographs AP, mortise, and lateral radiographs of the right ankle show tibiotalar osteoarthritis with varus hindfoot and talar tilt.

The right ankle radiographs show tibiotalar osteoarthritic changes with varus hindfoot and talar tilt (Figure 2).

**Figure 3:**
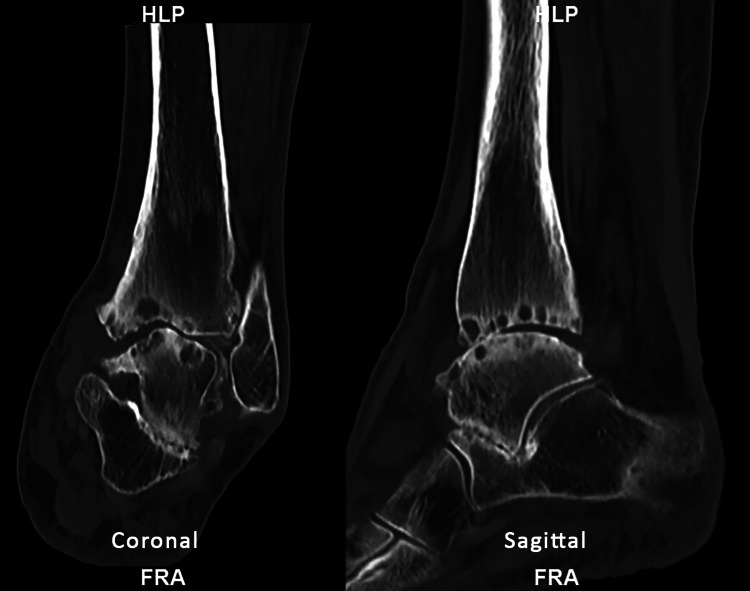
Coronal and sagittal CT scans of the left ankle Coronal and sagittal CT scans of the left ankle demonstrate significant osteoarthritic changes with subchondral cysts noted on tibia, talus, and calcaneus with sclerosing of the tibiotalar and subtalar joint surfaces. There is varus hindfoot and talar tilting noted.

To further delineate these pathologies, we obtained computed tomography (CT) scan of the left ankle. The CT scan demonstrated end-stage tibiotalar joint osteoarthritis with extensive subchondral cystic changes and significant varus hindfoot deformity along with significant subtalar osteoarthritis (Figure 3).

Given the findings on physical examination and imaging studies, we recommended a staged procedure to include bilateral tibiotalocalcaneal fusions utilizing an intramedullary device to the patient. We based this recommendation on the necessity to provide the patient with some measure of pain relief, realign her hindfoot, and provide a stable ankle upon which she could ambulate to improve her quality of life. The patient has elected to continue conservative management with planned surgery to follow at a different institution as the patient will be moving to a different state and will be able to have long-term follow-up. She is currently under the care of multiple medical subspecialties especially endocrinology and was started on teriparatide to treat her osteoporosis.

## Discussion

Strontium toxicity is quite rare and can evolve from environmental exposure during glass and ceramic making, municipal landfill operations, scrap metal sorting, and metal melting. It exists naturally in the soil in varying amounts and concentrations. Ingestion is the largest source of exposure through food and drinking water. Researchers have described iatrogenic toxicity, which is commonly associated with overdosing strontium vitamin supplementation or strontium ranelate, previously used to treat osteoporosis and osteoporosis adverse events [5]. Once absorbed by the gastrointestinal tract through passive diffusion, strontium is preferentially deposited and stored in human bone and teeth [2]. Similar to calcium, strontium impacts bone health through inhibition of bone resorption and improving mechanical properties of bone, thereby reducing risks of osteoporotic fractures [3].

Despite its potential benefit to bone health, high levels of strontium can negatively impact bone mineralization through a dose-dependent effect. Low doses of strontium stimulate bone formation and improve bone integrity by increasing BMD, but high concentrations decrease bone resorption and promote hypomineralization, ultimately decreasing the total calcium concentration in bone [6]. Strontium is excreted primarily through the kidneys and with time, it may accumulate in the blood leading to systemic symptoms such as bone marrow toxicity, renal impairment, cognitive impairment, vision changes, thyrotoxicity, and cardiomyopathy [7].

Researchers have studied the effects of strontium on bone. Earlier in vitro studies and clinical research demonstrated low circulating levels of strontium increased BMD. Meunier et al. conducted a large double-blinded randomized control trial regarding strontium ranelate treatment for osteoporosis. They demonstrated that low levels of strontium ingested daily reduced the risk of new vertebral fractures by 49% at one year and by 41% over three years. Strontium ranelate also increased BMD in the lumbar spine by 14.4 percent at three years [8]. The Treatment of Peripheral Osteoporosis Study (TROPOS) analyzed 5,091 postmenopausal women with osteoporosis in a double-blinded placebo-controlled study, which demonstrated that low-dose strontium ranelate treatments decreased the risk of non-vertebral fracture and vertebral fractures by 16% and 39%, respectively, over a three-year period [9].

Although low doses of strontium appear to have a positive effect on bone health, higher doses have the opposite effect. Verberckmoes et al. demonstrated the dose-dependent effects of strontium on osteoblast function and bone mineralization. In their in vitro study, low-dose strontium increased osteoblastic activity and BMD; however, high levels reduced mineralization indicating an inhibitory effect on hydroxyapatite formation within bone [10]. Increased levels of bone strontium were noted in dialysis patients and may play a role in renal osteodystrophy and ultimately leading to osteomalacia. Additional animal studies demonstrated high levels of strontium inhibited bone mineralization by reducing the intestinal absorption of calcium and inhibiting the conversion of 25(OH)D3 to 1,25(OH)2D3 [6]. 

We report a case of a patient with chronic strontium toxicity leading to significant osteoporosis and eventual end-stage tibiotalar osteoarthritis with varus deformity. To our knowledge, this is the first reported case. The patient will be moving to a different state and will continue with conservative measures. She will consider operative treatment to improve and restore her quality of life at a different institution where she will be able to have adequate long-term follow-up. From a surgical standpoint, our institution recommended staged bilateral tibiotalocalcaneal fusions secondary to end-stage subtalar and tibiotalar osteoarthritis and significant varus deformity. The patient did not want to consider a total ankle arthroplasty given her previous poor history with joint replacement surgery and metallosis. Other options include distraction arthroplasty and joint preserving deformity correction above the ankle joint. The patient was also treated with teriparatide per her endocrinologist and multi-disciplinary team.

## Conclusions

In conclusion, low doses of strontium can be beneficial to bone health and improve adverse events relating to osteoporosis. However, high doses of strontium can be detrimental leading to severe systemic complications. Although strontium toxicity is quite rare, this case demonstrates its deleterious effects on the quality of the bone leading to debilitating osteoarthritis and significant deformity in the foot and ankle. Metal toxicity can be a challenging diagnosis; therefore, clinicians should be aware of strontium toxicity and its systemic effects and take a multi-disciplinary approach to treatment for optimal management.
